# Impact of different headgear on release of bacteria when wearing a clean air suit for surgery: an experimental study

**DOI:** 10.1016/j.infpip.2026.100530

**Published:** 2026-03-12

**Authors:** B. Ljungqvist, B. Reinmüller, A. Tammelin

**Affiliations:** a Building Services Engineering, Chalmers University of Technology, Gothenburg, Sweden; b Department of Medicine, Solna, Unit of Infectious Diseases, Karolinska Institutet, Stockholm, Sweden

**Keywords:** Headgear, Disposable cap, Disposable hood, Reusable hood, Microbial air cleanliness

## Abstract

**Background:**

Staff wearing clean air suits made from tight material affects the level of bacteria in the air in operating rooms. This study investigated the influence of different types of headgear on levels of colony-forming units (CFU) in the air. Wearing no headgear was compared with wearing a disposable cap or a disposable hood, and with results from a previous study with a reusable hood made from the same material as the clean air suit.

**Methods:**

Tests with five healthy males wearing a clean air suit and different types of headgear were performed in a dispersal chamber according to Annex E in European standard EN 13795-2:2019.

**Results:**

The mean source strength value (number of bacteria emitted per second from one person) was 7.3 CFU/s without headgear and 7.2 CFU/S with a surgical cap (*P* = 0.52). The mean source strength value obtained with a disposable surgical hood was 5.3 CFU/s, which was not significantly lower than that obtained without headgear (*P* = 0.057).

**Discussion:**

The poor performance of the disposable surgical headgear was likely an effect of both permeable material and design. The mean source strength value with a textile hood in the previous study was 1.0 CFU/s, which was significantly lower than the mean source strength value without headgear (*P* < 0.01).

**Conclusion:**

When high microbial cleanliness of the air in the operating room is required, staff should wear a clean air suit fulfilling the criteria in European standard EN 13795-2, and a hood made of the same material as the rest of the suit.

## Introduction

Since Lidwell *et al.* clarified the relationship between deep surgical site infection and the level of colony-forming units (CFU) in operating room air, many measures have been taken to reduce the number of CFUs [1]. These include ventilation, restriction of door openings, restriction of number of persons attending, and clothing made from tight material worn by operating room staff [2]. The influence of each measure co-operates with the others. The impacts of door openings and number of people present in the operating room were studied by Erichsen-Andersson *et al.*, who showed that both factors significantly increased the number of CFU/m^3^ in an operating room with displacement ventilation, but were insignificant in an operating room with laminar airflow [3]. Studies by Tammelin *et al.* have shown that it is possible to reduce the CFU level in operating rooms with turbulent mixing ventilation – in operating rooms with low and high air supply – by using a more occlusive scrub suit, whereas in an operating room with unidirectional airflow, low CFU levels were achieved with a less occlusive scrub suit [4,5].

Internationally agreed and implemented standards, such as those of the European Committee for Standardization and International Organization for Standardization, are a good method to obtain medical devices with high and comparable quality. Sometimes, fulfilment of the demands in a standard is not sufficient to differentiate between products. This was shown in a study by Ljungqvist *et al.* [6], which compared three clean air suits which fulfilled European standard EN 13795-2:2019. In that study, the three tested outfits were worn together with a reusable textile hood made from the same material as the blouse and trousers. The present study used the clean air suit denominated ‘B’ in the study by Ljungqvist *et al.* A picture of the clean air suit with reusable hood is available in the published study [6].

To the authors' knowledge, the influence of choice of headgear on CFU level has not previously been investigated together with a modern clean air suit. This study aimed to explore the influence of two different types of disposable headgear on the CFU level emitted by the wearer, and to compare the effect of these with wearing no headgear, and also with the results obtained with the reusable hood made from the same material as the clean air suit in the previous study. To obtain comparable results with minimum influence from the environment, the tests were performed in a dispersal chamber in accordance with Annex E in European standard EN 13795-2:2019 [7].

It was hypothesized that a significant difference in source strength (i.e. number of bacteria emitted per second from one person) would be seen between results obtained with no headgear and results obtained with a surgical hood.

## Methods

### Types of headgear

**Figure 1:**
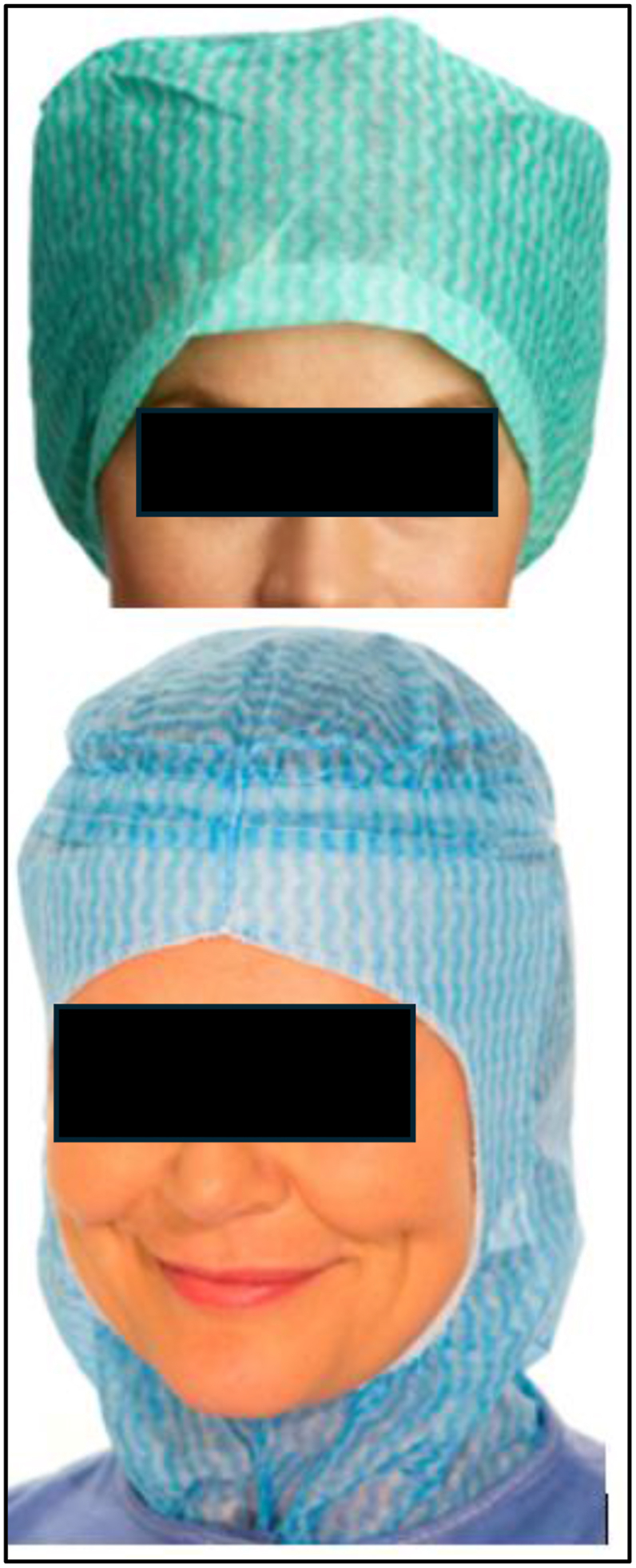
The two tested disposable headgears (shown on models).

The disposable surgical caps (Product No. 62100, Kosack; Mölnlycke, Gothenburg, Sweden) and disposable surgical hoods (Product No. 620205, Glenn; Mölnlycke) used in this study are shown in Figure 1.

### Test subjects

The test subjects were five healthy men, aged between 20 and 51 years, without any skin lesions.

### Dispersal chamber

A dispersal chamber is a qualified and validated chamber with a volume of approximately 2 m^3^, with tightly sealed walls and door, and a specified supply of high-efficiency particulate air (HEPA)-filtered air (inflow 220 L/s) at positive pressure (5 Pa) and controlled outflow. The design, technical data and function of the dispersal chamber used in this study (located at Chalmers University of Technology, Gothenburg, Sweden) were reported previously by Ljungqvist *et al.* and Lytsy *et al.* [6,8].

Bacteria present in the exhaust air from the dispersal chamber were grown on agar plates containing tryptic soy agar. After incubation, the number of CFUs was counted, characterized, and recorded as aerobic CFU/m^3^. The method is described in European standard EN 17141:2020 [9].

### Performance of test

**Figure 2:**
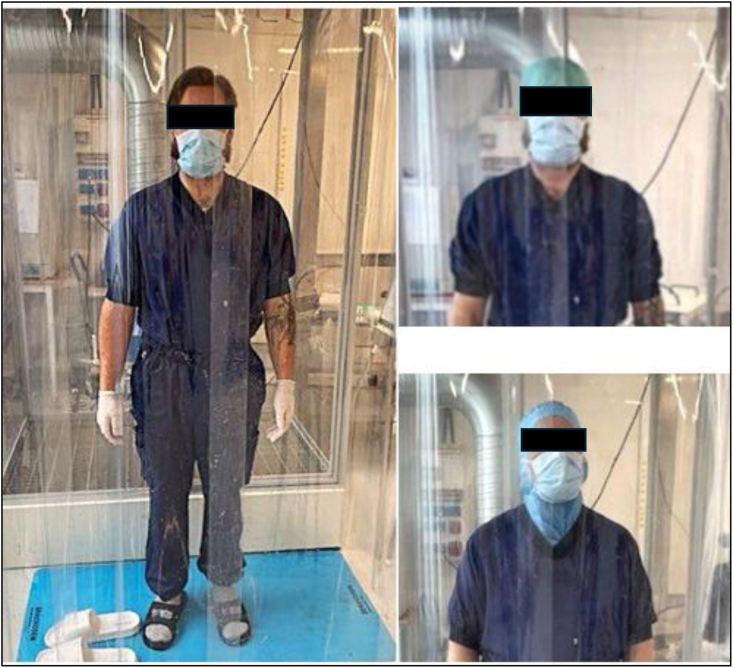
A test person without headgear standing outside the test chamber (left), and to the test persons with a disposable cap (top right) and disposable hood (bottom right).

The test subjects wore newly washed reusable clean air suits (Teximed Care; Almedahls, Kinna, Sweden) fulfilling the requirements for ‘high performance’ in European standard EN 13795-2:2019 [7]. The clean air suits were packed separately in sealed plastic bags. Additionally, subjects wore a disposable facemask; non-sterile gloves; washed but not new socks (65% cotton and 35% polyester); and clean, disinfected open plastic shoes (sandals) (Figure 2). Clothing and headgear were donned just before entering the dispersal chamber. The performance of standardized movements during the CFU measurements was the same as described by Ljungqvist *et al.* and Lytsy *et al.* [6,8]. The cycle of movements took 9 min, and was repeated four times without headgear and four times with each of the two headgears. Before each cycle of movements, the test subject stood still to avoid the influence of particle generation from the earlier test cycle. The dispersal chamber was cleaned between test subjects. All tests were performed during the same week in August 2025.

### Source strength

The source strength is described as the number of viable airborne particulates emitted per second from one person. By using the air volume in-flow in the dispersal chamber of 0.22 m^3^/s, in combination with the measured CFU amounts, the source strength for each test cycle was calculated.

The mathematical formula for calculating source strength is:

$$q_{S}=c\;\cdot \;Q$$

where q_s_ is source strength [bacteria-carrying particles (CFU/s)], c is concentration [bacteria-carrying particles (CFU/m^3^)], and Q is total air flow (m^3^/s).

### Data analysis

A two-sided Mann–Whitney *U*-test was used to compare the values for source strength obtained with subjects without headgear with the values obtained for each of the two tested headgears. Software provided by Statistics Kingdom (http://www.statskingdom.com) was used for analysis.

### Ethics

The recruitment of participants followed the Declaration of Helsinki. The study participants were able to terminate their participation in the study at any time.

## Results

Table I shows the source strength values (CFU/s) for every test subject and test cycle. The mean source strength value was 7.3 CFU/s without headgear and 7.2 CFU/S with a surgical cap. The difference was not significant (*P* = 0.52). The mean source strength value obtained with a disposable surgical hood was 5.3 CFU/s, which was not significantly less than that obtained without headgear (*P* = 0.057).

**Table I:** Source strength [colony-forming units (CFU)/s] calculated for each test cycle with test subjects lacking headgear, and for test subjects wearing a disposable surgical cap or a disposable surgical hood

Test subject: Test cycle	Source strength, CFU/s		
	Without any headgear	Disposable surgical cap	Disposable surgical hood
1:a	a	a	1.8
1:b	a	5.7	6.6
1:c	10.1	14.1	10.6
1:d	5.7	10.6	9.7
2:a	14.5	7.9	2.6
2:b	7.9	5.3	4.0
2:c	6.2	5.3	7.0
2:d	6.6	6.2	6.6
3:a	4.8	7.9	4.8
3:b	9.2	11.4	4.0
3:c	7.9	5.3	7.0
3:d	5.7	4.8	3.5
4:a	5.3	4.4	a
4:b	1.3	5.3	a
4:c	9.2	4.8	5.3
4:d	3.5	3.7	3.5
5:a	7.0	12.3	a
5:b	6.6	6.6	a
5:c	13.2	10.1	3.7
5:d	6.6	4.4	4.8
Mean value CFU/s	7.3	7.2	5.3
Min/max value	1.3/14.5	3.7/14.1	1.8/10.6

a No data available.

## Discussion

Wearing a cap or hood that fully covers hair on the head when entering an operating room has been routine practice worldwide for many years, and was recommended by the Centers for Disease Control and Prevention in 1999 [10]. It has been accepted implicitly as a measure to prevent hair from falling on surfaces, including the sterile field around the surgical wound and sterile instruments (i.e. part of contact prevention). It remains under debate whether headgear is also part of the prevention of airborne transmission of bacteria. Humphreys *et al.* found no reduction in the air counts of bacteria when comparing results from sampling with test persons without headgear and test persons wearing surgical hoods [11]. On the other hand, Friberg *et al.* found a significant increase in air counts and surface contamination near the surgical wound when headgear was omitted [12]. In 2002, a Working Party on Infection Control in the Operating Theatres established by the Hospital Infection Society launched recommendations on routines in the operating room [13]. The recommendation was that headgear should be worn by scrubbed staff alone during most operations, and was unnecessary for the non-scrubbed members of the operating team. However, during prosthetic implant operations performed in laminar flow theatres, it was recommended that all staff present in the operating room should wear headgear. This can be seen as an effort to minimize the risk of wound contamination during the most sensitive surgical procedures, but there were no arguments concerning CFU levels, which were presumably lower in operating rooms with laminar flow ventilation compared with operating rooms with other types of ventilation. In the mid-2010s, there was intense debate about recommendations by the Association of Perioperative Registered Nurses concerning surgical headgear. The American College of Surgeons questioned the recommendation that bouffant hats were superior to skull caps as they covered more of the wearer's hair [14]. A lower incidence of surgical site infection could not be shown by using bouffant hats [15,16]. Markel *et al.* compared the permeability, penetration and thickness of disposable bouffant hats, disposable skull cap hats and cloth skull caps, and found that disposable bouffant hats were not superior to disposable skull caps in terms of microbial contamination of surfaces and air in the operating room [17]. The cloth hats were less permeable than both types of disposable hat, but Markel *et al.* did not detect any differences in microbial shedding between any type of hat [17]. The poor protective capacity of the disposable surgical cap used in the present study, which resembled that tested by Merkel *et al.*, was probably an effect of both permeable material and design. A clean air suit made from a material with low permeability and designed with cuffs at arms, neck, waist and wrists has a weak point, namely the neckline. A hood which is tucked down at the neckline of the blouse should prevent skin scales from escaping this way. Nevertheless, the present results obtained with the disposable surgical hood were not significantly better than those obtained with the disposable surgical cap. The design of disposable headgear thus seems to have less impact than the permeability of the material.

In the study by Ljungqvist *et al.*, five male test persons performed the same tests in the dispersal chamber as described above, wearing the same type of clean air suit but with a reusable textile hood made from the same material as the blouse and trousers [6]. The mean source strength with the textile hood was 1.0 CFU/s (range 0.3–2.9) for the male participants, which was significantly lower than the results obtained without headgear in this study (*P* < 0.01).

Replacing disposable surgical headgear with reusable products can also have a positive effect on the climate footprint of surgery, as pointed out by Agarwal *et al.* [18].

### Strength and limitations

A strength of this study is the standardized method of testing, which reduced the environmental influence; this would not have been possible in an operating room. A limitation is that the number of test persons was low; however, the number was recommended in European standard EN 13795-2. Another limitation is that only two types of disposable headgear were evaluated, although these are the types used most commonly in Swedish hospitals.

It is recommended that further studies should be undertaken to compare headgear during authentic surgical procedures with staff wearing clean air suits that meet the requirements for ‘high performance’ in European standard EN 13795-2.

In conclusion, when high microbial cleanliness of air in the operating room is required, staff should wear a clean air suit that meets the criteria in European standard EN 13795-2 and a hood made of the same material.

## CRediT authorship contribution statement

**B. Ljungqvist:** Writing – original draft, Methodology, Investigation, Formal analysis, Conceptualization. **B. Reinmüller:** Writing – original draft, Methodology, Investigation, Formal analysis, Conceptualization. **A. Tammelin:** Writing – original draft, Methodology, Formal analysis, Conceptualization.

## Funding source

This study was initiated and funded by the Health and Medical Services Administration in Region Stockholm, Sweden.

## Conflict of interest statement

None declared.

## References

[1] Lidwell O.M., Lowbury E.J.L., Whyte W., Blowers R., Stanley S.J., Lowe D. (1984). Infection and sepsis after operations for total hip or knee-joint replacement: influence of ultraclean air, prophylactic antibiotics and other factors. J Hyg.

[2] World Health Organization (2018).

[3] Erichsen Andersson A., Petzold M., Bergh I., Karlsson J., Eriksson B.I., Nilsson K. (2014). Comparison between mixed and laminar airflow systems in operating rooms and the influence of human factors: experiences from a Swedish orthopedic center. Am J Infect Control.

[4] Tammelin A., Ljungqvist B., Reinmüller B. (2013). Single-use surgical clothing system for reduction of airborne bacteria in the operating room. J Hosp Infect.

[5] Tammelin A., Kylmänen P., Samuelsson A. (2023). Comparison of number of airborne bacteria in operating rooms with turbulent mixing ventilation and unidirectional airflow when using reusable scrub suits and single-use scrub suits. J Hosp Infect.

[6] Ljungqvist B., Reinmüller B., Tammelin A. (2023). Source strengths obtained with three clean air suits fulfilling the requirements for high performance in EN 13795-2:2019: an experimental study. Textil Res J.

[7] EN 13795-2:2019 (2019).

[8] Lytsy B., Hambraeus A., Ljungqvist B., Ransjö U., Reinmüller B. (2022). Source strength as a measurement to define the ability of clean air suits to reduce airborne contamination in operating rooms. J Hosp Infect.

[9] EN 17141:2020 (2020).

[10] Mangram A.J., Horan T.C., Pearson M.L., Silver L.C., Jarvis W.R., Hospital Infection Control Practices Advisory Committee (1999). Guideline for prevention of surgical site infection, 1999. Infect Control Hosp Epidemiol.

[11] Humphreys H., Russell A.J., Marshall R.J., Ricketts V.E., Reeves D.S. (1991). The effect of surgical theatre head-gear on air bacterial counts. J Hosp Infect.

[12] Friberg B., Friberg S., Östensson R., Burman L.G. (2001). Surgical area contamination – comparable bacterial counts using disposable head and mask and helmet aspirator system, but dramatic increase upon omission of head-gear: an experimental study in horizontal laminar air-flow. J Hosp Infect.

[13] Woodhead K., Taylor E.W., Bannister G., Chesworth T., Hoffman P., Humphreys H. (2002). Behaviours and rituals in the operating theatre. A report from the Hospital Infection Society Working Party on Infection Control in Operating Theatres. J Hosp Infect.

[14] Fabre V., Rock C., Abashian A., Trexler P., Maragakis L. (2018). Hats on: why hair must be covered, an infection prevention perspective. J Am Coll Surg.

[15] Kothari S.N., Anderson M.J., Borgert A.J., Kallies K.J., Kowalski T.J. (2018). Bouffant vs skull cap and impact on surgical site infection: does operating room headwear really matter?. J Am Coll Surg.

[16] Shallwani H., Shakir H.J., Aldridge A.M., Donovan M.T., Levy E.I., Gibbons K.J. (2018). Mandatory change from surgical skull caps to bouffant caps among operating room personnel does not reduce surgical site infections in class I surgical cases: a single-center experience with more than 15 000 patients. Neurosurgery.

[17] Markel T.A., Gormley T., Greeley D., Ostojic J., Wise A., Rajala J. (2017). Hats off: a study of different operating room headgear assessed by environmental quality indicators. J Am Coll Surg.

[18] Agarwal D., Bharani T., Armand W., Slutzman J.E., Mullen J.T. (2023). Reusable scrub caps are cost-effective and help reduce the climate footprint of surgery. Langenbecks Arch Surg.

